# Supplementary material for: Molecular Genomic Analyses of Enterococcus cecorum from Sepsis Outbreaks in Broilers
Source: Microorganisms. 2024 Jan 25;12(2):250. doi: 10.3390/microorganisms12020250 (PMC10892122; doi:10.3390/microorganisms12020250)
Supplement: Supplementary file 1 [file microorganisms-12-00250-s001.zip › Figure S1.pdf]

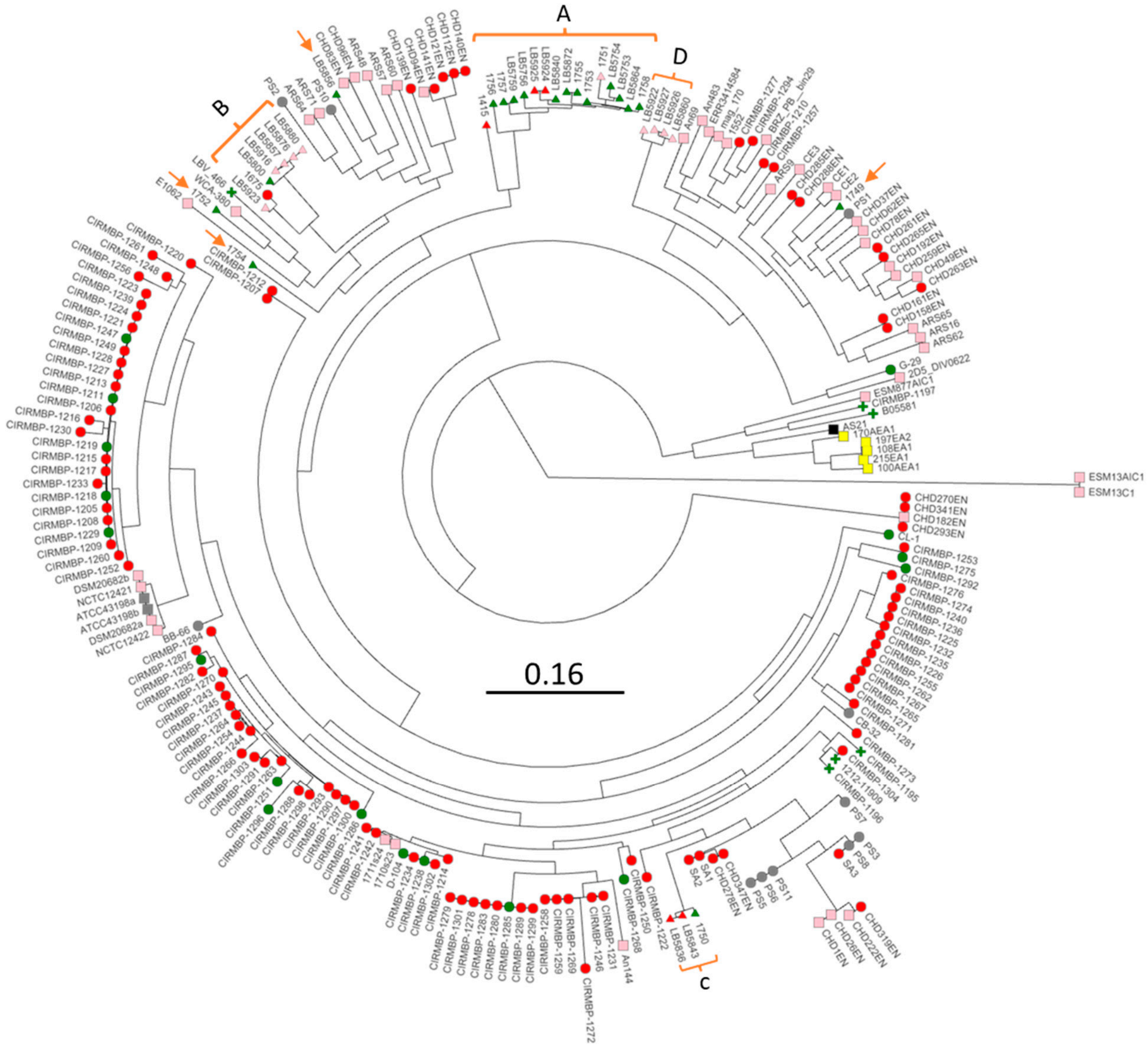

Figure S1. ParSNP phylogenomic tree of 227 *Enterococcus cecorum* isolates midpoint-rooted with *E. cecorum* SA1 as the reference genome. Strain names are the leaves. Clusters of USA isolates from the sepsis survey are bracketed with letters denoting cluster (Table 1), and orange arrows indicating solo isolates (as for Figure 1). Node colors indicate isolation anatomical location: Pink- air sac, intestine, cloaca, feces, egg residue, feces, rinsate, swab; Green- blood, heart, liver, spleen, peritoneum; Red- air sacculitis, bone marrow, leg joint, spine, osteomyelitis; Gray- unknown; Yellow- meat; Black- reactor. Node shape indicates the isolate source: Circle- UA poultry research farm, osteomyelitis, research clinic; Triangle- corporate survey; Plus- human; Square- not available/unknown.
